# Pharmacological Activities of Natural Products through the TGF-*β* Signalling Pathway

**DOI:** 10.1155/2022/9823258

**Published:** 2022-04-11

**Authors:** Chunpeng (Craig) Wan, Muhammad Farrukh Nisar, Hua Wu

**Affiliations:** ^1^Jiangxi Key Laboratory for Postharvest Technology and Nondestructive Testing of Fruits & Vegetables, College of Agronomy, Jiangxi Agricultural University, Nanchang 330045, China; ^2^Department of Physiology and Biochemistry, Cholistan University of Veterinary and Animal Sciences (CUVAS), Bahawalpur 63100, Pakistan; ^3^Department of Pathology, Medical College of Soochow University, Suzhou, China

Transforming growth factor-*β* (TGF-*β*) belongs to the TGF superfamily, which is composed of TGF-*β*s and other related proteins, such as bone morphogenetic proteins (BMPs) and activins. By binding to a complex of receptor serine/threonine kinases at the cell surface, the ligands bind to the TGF-*β* receptors and mainly activate the phosphorylation of Smad proteins and promote their translocation into the nucleus with a common Smad4, followed by the transcriptional activation of target genes. In addition to the Smad-dependent pathway, the activated TGF-*β* receptors can also activate other signaling pathways, such as mitogen-activated protein kinase (MAPK) signaling pathway [1]. Activation of TGF-*β* signaling has diverse intracellular biological functions, including cell differentiation, cell proliferation, and migration, dependent on the downstream effectors. For example, in the context of carcinoma, TGF-*β* signaling suppresses tumor growth through induced cell cycle arrest and increased apoptosis but facilitates tumor cell dissemination *via* the production of prometastatic factors and epithelial-to-mesenchymal transition (EMT) in cancer cells. Therefore, TGF-*β* signaling and the corresponding cellular response are regarded as both a tumor-suppressive and a tumor-supportive event during cancer development and progression [2].

Based on the findings of TGF-*β* signaling and its regulatory effect in physiological and pathological processes, targeting of TGF-*β* signaling has been studied and applied in various disease diagnoses. Inhibition of the TGF-*β*1 and its downstream signaling has been reported to suppress the activity of myofibroblasts and excessive synthesis of extracellular matrix in the kidney, which significantly limits renal fibrosis during the progression of chronic kidney disease (CKD) [3]. Recently, targeting at the extracellular domain of TGF-*β*-neutralizing TGFBR2 can block the TGF-*β* signaling in T helper 2 (TH2)-cell and remodel the microenvironment of tumor cells in breast cancer [4]. Pharmacological blockade of TGF-*β* signaling is recognized as a potential therapeutic approach for the treatment of chronic fibrosis and different tumor entities.

This special issue provides a comprehensive study of natural products and herbal medicine targeting at TGF-*β* signaling pathway in a diversity of chronic diseases and cancers. For the special issue, the editorial office accepted six manuscripts for publication after peer-review process and selected four original research articles and two review articles with outstanding quality. The four original articles focused on the topic of this special issue, which is the pharmacological effect of natural products or herbal medicine targeting at the TGF-*β* signaling pathway. One review article emphasizes on the role of TGF-*β* signaling pathway and development of targeted therapy, while the other review provides an overview of the therapeutic effect of natural polyphenolic flavanone on the treatment of chronic diseases and malignancies.

The original paper “Antitumor Effects of Baicalein and Its Mechanism via TGF*β* Pathway in Cervical Cancer HeLa Cells” by G. Yu reported the therapeutic effect of baicalein, which was isolated from the Chinese herb of *Scutellaria baicalensis*, on the treatment of cervical cancer. The article illustrates the activity of baicalein on TGF-*β* signaling pathway blockage, overexpression of cell-adhesion molecule E-cadherin, and inhibition of EMT process in cervical cancer HeLa cells, which demonstrates the antimigratory effect of baicalein on cervical cancer. The original paper “Ginsenoside Rb3 Alleviates the Toxic Effect of Cisplatin on the Kidney during Its Treatment to Oral Cancer via TGF-*β*-Mediated Mitochondrial Apoptosis” by W. Wu assessed the activity of ginsenoside Rb3 in the oral cancer patients with cisplatin treatment. This article identified the protective effect of Rb3 on the activation of TGF-*β* signaling pathway and cellular apoptosis of kidney cells, determined by the cleavage of PARP and caspases driven by cisplatin administration, which consequently revealed the advantage of Rb3 pretreatment before chemotherapy in oral cancer patients. The research article “Punicalin Alleviates OGD/R-Triggered Cell Injury via TGF-*β*-Mediated Oxidative Stress and Cell Cycle in Neuroblastoma Cells SH-SY5Y” by T. Yang identified an active component from *Punica granatum* L. (punicalin) and analyzed its protective effect on oxidative stress of cells followed by TGF-*β* signaling activation during ischemia/reperfusion injury. In addition, the article “Study on Network Pharmacological Analysis and Preliminary Validation to Understand the Mechanisms of Plantaginis Semen in Treatment of Gouty Nephropathy” by H. Zhao screened for potential targets from public online databases and identified Plantaginis Semen as a potential inhibitor of TGF-*β* signaling in gouty nephropathy (GN). Activity of Plantaginis Semen was confirmed by the analysis of inflammatory factors, including TGF-*β*1, TNF-*α*, and IL-1*β*, as well as urinary protein levels in the treatment of GN.

In the review article “The Role of TGF-*β* Signalling Pathways in Cancer and Its Potential as a Therapeutic Target” by Y. Yang, the authors comprehensively discussed the multiple functions of TGF-*β* signaling and downstream effectors in different stages of tumorigenesis. The article gives an overview of the tumor-suppressive role of TGF-*β* signaling through the cell cycle arrest and tumor cell apoptosis and, meanwhile, the prometastasis activity of TGF-*β* signaling in the reestablishment of tumor-favorable microenvironment. Moreover, the review article also summarized the therapeutic components targeting at TGF-*β* family ligands or TGF-*β* receptors, including antisense oligonucleotides and monoclonal antibodies. The other review article “Pharmacological Activity of Eriodictyol: The Major Natural Polyphenolic Flavanone” by Z. Deng gives insight into the flavonoid eriodictyol with negative regulatory effect of PI3K/Akt signaling pathway, matrix metalloproteinases (MMPs) expression, and increased cell apoptosis, which highlights the therapeutic effect of eriodictyol in the protection of cell injury as well as the treatment of malignant tumors.
